# Thalidomide and Phosphodiesterase 4 Inhibitors as Host Directed Therapeutics for Tuberculous Meningitis: Insights From the Rabbit Model

**DOI:** 10.3389/fcimb.2019.00450

**Published:** 2020-01-14

**Authors:** Ranjeet Kumar, Afsal Kolloli, Pooja Singh, Christopher Vinnard, Gilla Kaplan, Selvakumar Subbian

**Affiliations:** ^1^New Jersey Medical School, Rutgers, Public Health Research Institute, The State University of New Jersey, Newark, NJ, United States; ^2^University of Cape Town, Cape Town, South Africa

**Keywords:** tuberculosis, mycobacterium, cerebrospinal fluid, phosphodiesterase-4 inhibitors, inflammation, corticosteroid, tumor necrosis factor-alpha

## Abstract

Tuberculous meningitis (TBM) is the most devastating form of extrapulmonary *Mycobacterium tuberculosis* infection in humans. Severe inflammation and extensive tissue damage drive the morbidity and mortality of this manifestation of tuberculosis (TB). Antibiotic treatment is ineffective at curing TBM due to variable and incomplete drug penetration across the blood-brain barrier (BBB) and blood-cerebrospinal fluid (CSF) barriers. Adjunctive corticosteroid therapy, used to dampen the inflammation, and the pathologic manifestation of TBM, improves overall survival but does not entirely prevent the morbidity of the disease and has significant toxicities, including immune-suppression. The rabbit has served as a fit for purpose experimental model of human TBM since the early 1900s due to the similarity in the developmental processes of the brain, including neuronal development, myelination, and microglial functions between humans and rabbits. Consistent with the observations made in humans, proinflammatory cytokines, including TNF-α, play a critical role in the pathogenesis of TBM in rabbits focusing the attention on the utility of TNF-α inhibitors in treating the disease. Thalidomide, an inhibitor of monocyte-derived TNF-α, was evaluated in the rabbit model of TBM and shown to improve survival and reduce inflammation of the brain and the meninges. Clinical studies in humans have also shown a beneficial response to thalidomide. However, the teratogenicity and T-cell activation function of the drug limit the use of thalidomide in the clinic. Thus, new drugs with more selective anti-inflammatory properties and a better safety profile are being developed. Some of these candidate drugs, such as phosphodiesterase-4 inhibitors, have been shown to reduce the morbidity and increase the survival of rabbits with TBM. Future studies are needed to assess the beneficial effects of these drugs for their potential to improve the current treatment strategy for TBM in humans.

## Introduction

*Mycobacterium tuberculosis* (Mtb), the causative agent of tuberculosis (TB), is transmitted via aerosols exhaled by patients with active disease and acquired by new contacts via inhalation of infectious droplets, resulting in the establishment of infection in the lungs. However, the bacteria can be transported by infected macrophages/dendritic cells from the lungs to other organs, such as lymph nodes, the spine, and the brain, resulting in disseminated extrapulmonary disease (Krishnan et al., 2010). Tuberculous meningitis (TBM), a common manifestation of Mtb-infection of the central nervous system (CNS), is associated with non-suppurative inflammation of the dura mater and meninges. TBM constitutes ~5–10% of all extrapulmonary TB cases and thus represents less than 1% of all active TB cases. However, it is the deadliest form of TB with frequent mortality and severe morbidity (Farer et al., 1979; Torok, 2015). TBM is most commonly seen in children under the age of 4 years and older people. In addition, immune-suppression such as that seen in HIV-infection is associated with a significantly higher frequency of Mtb dissemination from the lungs to extrapulmonary sites, including the CNS (Dube et al., 1992; Thwaites et al., 2005; Marais et al., 2010; Ducomble et al., 2013; Heemskerk et al., 2016). A recent meta-analysis of the global TBM burden has reported about a 40% mortality rate within 6 months of TBM diagnosis with progressively deteriorating disease (van Laarhoven et al., 2019).

## Clinical Symptoms of Human TBM

Following Mtb dissemination and establishment of infection in the CNS, slow-progressive meningitis develops with symptoms such as headache, fever, vomiting, and neck stiffness, which are not easily distinguishable from other forms of meningitis (Wilkinson et al., 2017). If untreated, TBM progresses to more severe clinical symptoms such as unconsciousness, focal neurological deficits, seizures, raised intracranial pressure, hemiparesis, and cranial nerve palsies. Many of these symptoms are driven by the exacerbated inflammatory response to Mtb infection and mediated by the cytokines released into the CNS from infected immune cells, including TNF-α (Davis et al., 2019). In ~50% of patients with advanced TBM, the fifth and third cranial nerves are affected, and about 10% of patients report limb weakness (either hemiplegia or paraplegia). At this stage, death is almost inevitable in the absence of any therapeutic interventions (Dastur et al., 1995; Thwaites et al., 2000). Key laboratory findings in the CSF of patients with TBM include pleocytosis with lymphocyte predominance (150–1,000 leukocytes/μl with a mixed population of neutrophils and lymphocytes), high protein content (0.8–2.0 g/dl) and low glucose levels (CSF: plasma glucose ratios of <0.5). TBM in patients with HIV co-infection is characterized by the absence of mononuclear leukocytes and/or the presence of a large number of neutrophils (>1,000 cells/μl), mimicking acute pyogenic bacterial meningitis (Torok, 2015). Elevated levels of inflammatory cytokines are commonly seen in the CSF and plasma of patients with TBM. Radiographic features in TBM patients include basal meningeal exudates, infarction, tuberculomas, and hydrocephalus.

## The Current Strategy for TBM Treatment

The current TBM treatment strategy includes concurrent administration of standard anti-TB drugs and corticosteroid (Uhlin et al., 2012). Even though antibiotic therapy reduces mortality among TBM cases, the poor penetration of the drugs across the BBB and the associated exacerbation of the inflammatory response to the antibiotic-mediated killing of Mtb results in the inefficient cure of TBM. Since clinical manifestations of TBM are primarily driven by severe inflammation in the confined space of the cranium, anti-inflammatory drugs, such as corticosteroids, have been widely used as adjunctive therapy (Wasserman et al., 2019). Several studies, including a randomized-controlled clinical trial, have reported that treatment with corticosteroid helps to control exacerbated inflammation in TBM cases (Thwaites et al., 2004). The combination therapy of standard anti-TB drugs, along with dexamethasone, has been shown to prolong the survival and reduce mortality among HIV-negative TBM cases (Prasad et al., 2016). However, no significant difference was noted in severity of vasculitis among TBM cases treated with adjunctive corticosteroids compared to the placebo-treated cases (Schoeman et al., 1997). Moreover, adjunctive corticosteroid with antibiotic therapy did not affect the elevated levels of inflammatory cytokines such as TNF-α in the CSF of TBM patients (Donald et al., 1995).

Contradictory findings have been published on the utility of corticosteroids to reduce TBM-associated disability in patients (Nguyen et al., 2007). In some of these studies, a high dose of adjunctive corticosteroid administration controlled the inflammation and reduced other neurological complications associated with TBM in 50% of patients (Prasad et al., 2016). In other studies, adjunctive corticosteroid treatment did not alter or even worsened the clinical condition of the patients and failed to prevent neurological disability associated with TBM (Garg et al., 2014). These findings underlie the necessity of exploring additional adjunctive anti-inflammatory drugs, which can better dampen excessive inflammation caused by Mtb and provide beneficial effects when combined with antibiotics, as host-directed therapy for TBM.

## Alternative Anti-Inflammatory Drugs for TBM Treatment

The limited efficacy of the standard use of corticosteroids as adjunctive therapy for TBM patients has led to attempts to identify alternative, improved host-directed therapeutic (HDT) treatment modalities for this disease. Thus, aspirin and thalidomide, both known to have anti-inflammatory properties, have been tested for their ability to control pathogen-mediated inflammation in TBM (Uhlin et al., 2012).

One of the clinical manifestations of TBM in children is the development of arterial stroke, which is associated with poor outcome of this disease (Schoeman et al., 2011). Aspirin, an anti-inflammatory, anti-thrombotic, and anti-ischemic drug, was evaluated for its potency in the treatment of arterial stroke associated with pediatric TBM. Data from a randomized, open-label clinical study conducted with 118 TBM patients in India show that adjunctive aspirin treatment (150 mg/day) significantly reduced the 3 month mortality rate (21.7%) and reduced the risk of stroke (19.1%), compared to the placebo-treated patients (Misra et al., 2010). However, no beneficial effect was found for aspirin treatment in another randomized clinical study conducted in South Africa with 146 pediatric TBM cases (Schoeman et al., 2011). In the South African study, the host-modulating functions of aspirin was dose-dependent, and all doses were well-tolerated. However, aspirin did not significantly reduce the morbidity of pediatric TBM patients (Schoeman et al., 2011). A meta-analysis that reviewed four randomized clinical studies totaling 546 TBM cases concluded that compared to the placebo-group, adjunctive aspirin treatment reduced the risk of new infarctions significantly, but not the mortality rate. The measurable adverse events were comparable between the placebo- and aspirin-treatment groups (Rizvi et al., 2019). However, the beneficial effect of adjunctive aspirin treatment in TBM cases with different levels of disease severity remains unclear.

Another anti-inflammatory drug that has been tested as an adjunctive to antibiotics for TBM treatment is thalidomide (α-N-[phthalimido] glutarimide). This non-barbiturate sedative, shown to be a potent antiemetic drug was widely prescribed for the treatment of morning sickness among pregnant women during the early 1960s. Although it became commonly used in Europe and Asia, the US Food and Drug Administration did not approve the sale of thalidomide in the US, due to concerns about the safety of the drug. This concern was later compounded by the finding that administration of thalidomide to pregnant women resulted in teratogenic effects, such as limb malformation in the newborns (Melchert and List, 2007). However, in the 90s, thalidomide treatment was shown to reduce TNF-α-associated inflammation significantly in patients with erythema nodosum leprosum (ENL) (Sampaio et al., 1991). Thereafter, a number of studies have confirmed these observations and demonstrated that ENL patients experience significantly improved clinical outcomes (Sampaio et al., 1993; Corral and Kaplan, 1999; Haslett et al., 2005; Millrine and Kishimoto, 2017).

The TNF-α inhibitory capacity of thalidomide prompted the evaluation of the drug's ability to control the inflammation in the CNS during TBM (Schoeman et al., 2000). In general, results from these studies suggest that thalidomide has a beneficial effect on TBM during antibiotic treatment in children and adults (Schoeman et al., 2000; Roberts et al., 2003). A dose-escalation study reported that administration of 6–24 mg/kg/day of thalidomide was well-tolerated among children suffering from stage-2 TBM (Schoeman et al., 2010). In contrast to treatment with anti-TB drugs with/without adjunctive corticosteroid treatment, pediatric TBM cases treated with adjunctive thalidomide showed significant improvement in disease manifestations; they did not develop new infarcts, as evidenced by CT scan analysis, and showed resolution of basal exudate. Moreover, thalidomide-treated TBM patients showed significantly reduced TNF-α levels in the CSF (Schoeman et al., 2010). Clinical case studies in pediatric meningitis have also demonstrated beneficial effects of thalidomide. A case-study of adjunctive thalidomide administration (4–5 mg/kg for 3–8 months) reported remarkable clinical recovery in four children suffering from TBM-related optochiasmatic arachnoiditis, which is a consequence of intracranial pressure and/or inflammation of the basal arachnoid (Schoeman et al., 2010). A double-blind, randomized clinical study of adjunctive high dose thalidomide treatment (24 mg/kg/day for 1 month) in stage-2 and -3 TBM cases did not significantly improve treatment efficacy, compared to the placebo group (Schoeman et al., 2004). However, in this study, the TBM cases in the adjunctive thalidomide treatment group had higher disease severity at baseline, compared to the placebo group (Schoeman et al., 2004). Recently, two clinical case studies revealed the beneficial role of thalidomide in controlling the neuroinflammation observed during CNS TB. Administration of thalidomide, in combination with anti-TB drugs and corticosteroids, was shown to improve the clinical outcome of patients with chronic TBM symptoms (Keddie et al., 2018). Another case study reported that adjunctive thalidomide (1.2 mg/kg/day) therapy with standard anti-TB drugs (HRZ) and low dose corticosteroid significantly improved the clinical status of the patient, after 5 months of treatment (Caraffa et al., 2018). These observations suggest that thalidomide might improve patient responses to antibiotic treatment, especially where traditional high dose corticosteroid therapy fails to control chronic inflammation and other neurological complications associated with TBM.

Although thalidomide is effective in reducing inflammation associated with infection in such conditions as ENL, the potential of this drug to activate T-cells may interfere with the anti-inflammatory property of the drug (Haslett et al., 1998, 1999). Studies conducted using animal models, and human peripheral blood samples show that thalidomide can function as a co-stimulatory signal to activate T cells. This increase in T cell proliferation is associated with elevated production of IL-2 and IFN-γ by these cells. Thus, thalidomide treatment can contribute to the development of immune reconstitution inflammatory syndrome (IRIS) in pulmonary TB patients with or without co-infection with HIV (Haslett et al., 1998, 1999; Corral and Kaplan, 1999). Consequently, there is a need to develop alternative compounds that are safe, retain the anti-TNF-α activity of the parent drug, but do not activate T-cell mediated immunity. Future studies are required to evaluate new derivatives of thalidomide for their potential to change and/or to improve the current treatment strategy for TBM.

## The Rabbit as a Preclinical Model to Assess the Pathogenesis and New Therapies in TBM

**Figure 1:**
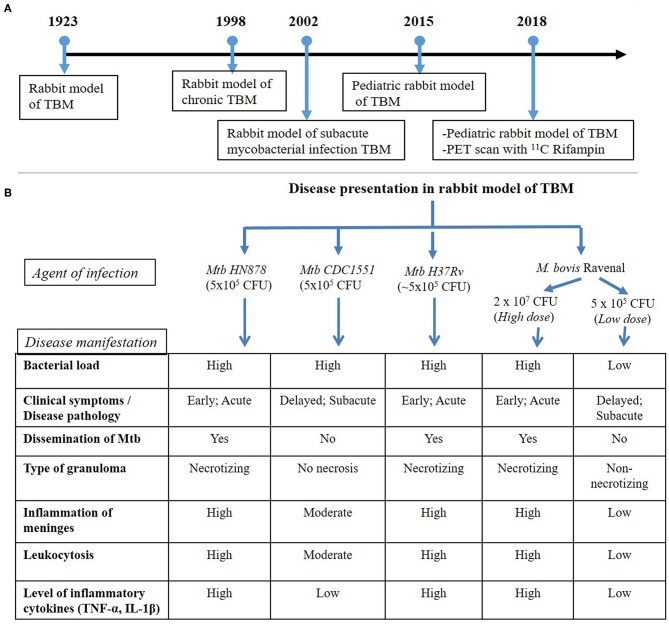
**(A)** Timeline of rabbit model TBM development. The first experimental model of rabbit TBM was established in 1923 using intra-cisternal inoculation of pathogenic mycobacteria. This model shows the presence of *Mycobacteria* in the meninges (Rich foci), disease progression with various infection/inoculum doses of different mycobacterial strains (chronic and subacute TBM) and the efficacy of anti-inflammatory drugs, such as thalidomide, on TBM. Since the TBM has a high mortality rate among children, a pediatric model of rabbit TBM was also developed and evaluated for disease progression in this population. Recent developments with the rabbit TBM model have introduced imaging techniques, such as PET scan (^124^*I*-*DPA*-*713*), which is vital in understanding host-drug interactions during TBM treatment. **(B)** Disease presentation in rabbit model of TBM. The rabbit model of TBM have used different strains of pathogenic *Mycobacterium*, which demonstrated heterogeneity in the outcome of infection. High- and low-inoculum doses differ in their onset of disease symptoms and the rate of progression of infection. The laboratory Mtb strain H37Rv, clinical Mtb isolate HN878 and a high dose *M. bovis* Ravenal infection led to elevated cytokine response and severe inflammation of the meninges. These animals had early and robust disease pathology, accompanied by the presence of necrotizing granulomas and dissemination of Mtb to other organs of the body. In contrast, infection by Mtb CDC1551 and a low-dose *M. bovis* Ravenal, displayed a delayed and moderate-to-low inflammatory response without bacillary dissemination to other organs of the infected animals.

The rabbit, as an experimental TBM model of human disease, was established in 1923 (Kasahara, 1924). The first TBM rabbit model involved the atlanto-occipital inoculation of human tubercle bacilli (presumably Mtb), avian tubercle bacilli, and bovine tubercle bacilli into the subarachnoid space (Figure 1). The inoculum, prepared in a saline solution, did not cause any initial visible reaction in the rabbits. However, at 6–15 days post-inoculation, tubercles had formed in the meninges, accompanied by signs of TBM, such as paralysis, followed by death. This early study demonstrated the resemblance of disease progression and immunopathology between human TBM and the rabbit model. The tubercles were mostly present at the base of the cerebrum and cerebellum, and less frequently on the spinal cord (Kasahara, 1924). This study also determined the chemical composition and physical appearance of CSF from rabbits with TBM and compared it to that of healthy animals (Table 1). Rabbits with TBM showed a decrease in CSF glucose content, an increase in protein levels and lymphocyte counts, and loss of anticoagulant properties. Clinical signs, such as loss of coordination, head tremor, rigidity of neck and back muscles, head retraction and ataxia, convulsions, and paralysis of the legs, marked the course of progressive disease pathology in rabbits with TBM (Kasahara, 1924). A classic study compared TBM disease pathology between children and the rabbit model, demonstrating that the presence of Mtb in the meningeal space directly leads to progressive TBM in both animals and humans (Rich and McCordock, 1933). Importantly, the clinical and CSF findings in the rabbit TBM model are very similar to human disease, with a predominance of lymphocytes, high protein levels, and viscous CSF consistency (Table 1). Moreover, basal meningeal enhancement and hydrocephalus are common abnormalities seen both in the rabbit model of TBM and in human TBM cases (Torok, 2015).

**Table 1:** Comparison of CSF characteristics between human and rabbit during normal and TBM conditions.

**Species**	**Normal**	**TBM**
Human	Clear and colorless	Opalescent, turbid and slightly yellow in color
	No Coagulation	Coagulation
	Total cell count: Adult- 0–5 cells/mm^3^ Infants- 0–30 cells/mm^3^	Total cell count: 10–1000 cells/mm^3^ (Mainly monocytes, lymphocytes predominate and neutrophils present in the early stages)
	Glucose: 45–85 mg/dl	Glucose: <45 mg/dl
	Protein: 15–45 mg/dl	Protein: 100–500 mg/dl
	Specific gravity: 1.004–1.007	
Rabbit	Clear and colorless	Turbid, cloudy and off-white in color
	No Coagulation	Coagulation
	Total cell count: 0–10 cells/mm^3^	Total cell count: 0–1500 cell/mm^3^ PMN: 80–400/mm^3^ Lymphocyte: 300–1200/mm^3^
	Protein- albumin: 0.015–0.019% Globulin: Absent	Protein: 0.03–0.8%
	Glucose: 0.054%	Glucose: 0.042–0.01%
	Specific gravity: 1.005	

Another similarity between humans and the rabbit model of TBM is the cellular response underlying brain infection by Mtb. Microglial cells play an essential role in eliciting an immune response against Mtb in the case of TBM (Curto et al., 2004; Rock et al., 2008). Infected human microglial cells produce cytokines and chemokines *in vitro*, including TNF-α, IL-6, IL-1β, CCL2, CCL5, and CXCL10 (Rock et al., 2008). A recent study reported elevated production of inflammatory cytokines, including TNF-α and IFN-γ in whole blood from TBM patients upon *in vitro* stimulation with Mtb (van Laarhoven et al., 2019). Similar to the human conditions, mycobacterial invasion of microglia cells/macrophages leads to the release of pro-inflammatory cytokines in the rabbit model of TBM (Tsenova et al., 1998).

Although TNF-α has a protective role against Mtb infection, increased levels of this proinflammatory cytokine in TBM exacerbate inflammation that contributes to tissue destruction, increased protein, and decreased glucose levels (Tsenova et al., 1999). In the rabbit model of TBM, administration of a recombinant human TNF-α impaired the transportation of compounds/drugs across the BBB into the brain, and led to CSF leukocytosis, protein influx, and lactate accumulation.

The rabbit model of TBM also displays Mtb strain-specific immunopathology, which can contribute to our understanding of protective vs. permissive host responses to infection. For example, intra-cisternal infection of rabbits with the clinical Mtb isolates HN878 vs. CDC1551, produces significantly different disease outcome (Tsenova et al., 2005). Severe disease manifestations with higher bacterial loads in the CSF and brain were evident after 8 weeks of intra-cisternal inoculation with the clinical Mtb strain HN878. These animals also had necrotizing granulomatous meningitis, encephalitis, and vasculitis within the cortex of the brain. Progressive meningitis led to the loss of coordination and limb paralysis by 3 weeks post-infection (Tsenova et al., 2005). In contrast, the bacterial load in the CSF of rabbits infected with the clinical Mtb isolate CDC1551 was significantly lower, with no bacterial dissemination to other organs after 7 days of infection. These differences disappeared by the end of 4 weeks post-inoculation. Elevated proinflammatory cytokine levels and high protein levels, as well as leukocytosis, were observed in the CSF upon the establishment of infection with both Mtb isolates. However, at 6 weeks post-infection, rabbits infected with the clinical Mtb isolate CDC1551 had reduced TNF-α levels, concomitant with a reduced bacterial count, and no clinical signs of disease progression. Similarly, histologic examination of brain tissue from CDC1551 infected animals confirmed the moderate inflammation of the meninges, limited leukocytic infiltration and no necrosis (Tsenova et al., 2005).

In addition to the nature of the infecting Mtb strain, the inoculum dose of pathogenic mycobacteria used for infection can also affect the clinical outcome of TBM. Using this approach, a subacute model of mycobacterial CNS infection, with a delayed onset of disease, was established (Tsenova et al., 2002). In this model, intra-cisternal inoculation of rabbits with 5 x 10^4^ CFU of *M. bovis* Ravenel induced progressive subacute meningitis characterized by high CSF leukocytosis, protein influx, and release of TNF-α, with substantial meningeal inflammation by 28 days post-infection. This rabbit model of subacute TBM was further refined by titrating different inoculum doses of Mtb and observing the temporal kinetics of bacterial growth and disease progression (Tsenova et al., 2002).

The developmental stages of the brain in children makes them highly vulnerable to TBM. To understand the pathogenesis of TB during early brain development, a pediatric rabbit model of TBM was created (Tucker et al., 2016). In this model, 4–8 day old NZW rabbits were inoculated with Mtb into the subarachnoid space, and the animals were monitored for 35 days post-infection. In these animals, disease symptoms appeared by the 7th day post-infection, characterized by neurologic manifestations of TBM, including inability of the animals to move and balance their body parts and loss of head and body elevation. Further, disease scores from the neurobehavioral testing of these animals suggested a gradual loss of motor function and decreased maturity associated with progressive disease. Activation of microglia, the primary cells in brain that harbor Mtb during infection was tracked using a PET radiotracer for macrophage-associated inflammation (^124^*I*-*DPA*-*713*) and confirmed with the presence of Iba-1, a marker of microglia. A PET-imaging study of pediatric rabbits with TBM revealed localization of the tracer around TB lesions in the brain and the site of injection at the 21st-day post-infection. In these animals, enlargement of the TB lesion was noted with time. The bacterial burden in brains, with superficial medial tuberculoma, increased until 14 days of infection after which it remained stable. Gradual dissemination of Mtb into the lungs was observed until 21 days that became stable with no significant increase until 35 days post-infection. At this time, microscopic examination revealed deep tuberculomas with increased Mtb CFU.

The penetration of antibiotics across the BBB during a multi-drug treatment regimen was evaluated in the pediatric rabbit TBM model (Tucker et al., 2018). In this study, 4–8 days postnatal rabbits infected in the subarachnoid space with Mtb H37Rv were treated after 21 days of infection with a combination of ^11^C rifampin, isoniazid, and rifampicin for 6 weeks, followed by PET imaging. Results from this study revealed limited penetration of rifampin that diminished as early as 2 weeks post-treatment. PET scans also unveiled absence of antibiotics at the site of TB lesions (Tucker et al., 2018). Thus, a pediatric rabbit model of TBM infection provided a vital tool for our understanding of the effect of Mtb infection and disease progression on neuronal development, myelination, and microglia present in major white matter tracts, since these processes are very similar between rabbit and human brain development.

Taken together, the rabbit model of TBM recapitulates the pathologic manifestations seen in human disease. This model is useful to test therapeutic interventions after the development of clinical signs and to evaluate the effect of different regimens of anti-TB and immunomodulatory treatments on the course of the disease (Sampaio et al., 1991, 1993; Moreira et al., 1993; Tramontana et al., 1995; Tsenova et al., 2002).

Although the rabbit model recapitulates several clinical and pathologic manifestations of TBM in humans, it has some limitations. In the rabbit model, Mtb is inoculated directly into the intra-cisternal region. This may not reflect the natural dissemination that occur in patients with TBM. Similarly, disease manifestations of TBM in rabbits are associated with the nature of the mycobacterial strains and the infectious inoculum used. However such associations are not well-established in human patients. Therefore, future studies should fine-tune the rabbit and potentially other animal models of TBM, to mimic the natural route of Mtb transmission, without compromising the pathology of human disease. Nonetheless, experimental TBM in outbred rabbits displays the heterogeneity in host response, as seen in human patients (Tsenova et al., 2006).

## Discovery of Next-Generation Anti-Inflammatory Drugs as HDT for TB

During the last decade, several structural analogs of thalidomide have been synthesized and evaluated for their potency as selective TNF-α inhibitors (Corral et al., 1996; Muller et al., 1996, 1999; Gordon and Goggin, 2003). Ultimately, two classes of novel compounds with TNF-α inhibitory activity were identified: Selective Cytokine Inhibitory Drugs (SelCIDs) and Immunomodulatory Drugs (IMiDs) (Corral and Kaplan, 1999; Marriott et al., 2001). The IMiDs were shown to be more potent inhibitors of TNF-α than the parent drug thalidomide. These IMiDs, such as Lenalidomide, CC5013 (Revlimid) and CC4047 (Actimid) (Celgene Corp.) decreased TNF-α expression by about 2,000 to 50,000-fold. The efficacy of these compounds has been evaluated in hematologic malignancies, including myelodysplastic syndrome (MDS) and multiple myeloma (MM) (Bartlett et al., 2004; Millrine and Kishimoto, 2017). In addition to TNF-α, IMiDs inhibit the expression of diverse pro-inflammatory cytokines, including IL-1β, IL-6, and IL-12. Similar to thalidomide, IMiDs stimulate differentiation and proliferation of T-cells; treatment of human PBMCs with IMiD CC-4047 upregulated the expression of CD40 ligands on T-cells, co-stimulated T-cell proliferation, and induced IFN-γ and IL-2 expression (Keifer et al., 2001). The IMiDs were also shown to be teratogenic.

A rabbit study of sub-acute CNS mycobacterial infection, established by intra-cisternal injection of low-dose pathogenic *M. bovis*, was shown to recapitulate the pathophysiology of human TBM (Tsenova et al., 2002). In these animals, treatment with anti-TB drugs did not improve the clinical deterioration; nearly 50% of animals showed signs of progressive neurologic disease and death, although the bacillary load was reduced in the brain and in the CSF. Disease manifestations were associated with elevated TNF-α levels in the CSF. In contrast, treatment of rabbits with TBM with antibiotics plus adjunctive IMiD3 CC-5013 (lenalidomide) improved the clinical outcome and reduced mortality. The beneficial effect of IMiD3 has been attributed to its ability to dampen TNF-α, IL-1β, IL-6, and other inflammatory cytokines. Additionally, IMiD3 has been shown to cross the BBB effectively and accumulates in CSF at sufficient concentrations without interfering with the penetration of anti-TB drugs into the CSF (Tsenova et al., 2002). Thus, IMiDs may be promising adjunctive drugs in treating TBM in humans. However, the IMiDs, similar to thalidomide, are teratogenic and, therefore, may not be the treatment of choice for an infectious disease such as TBM.

**Figure 2:**
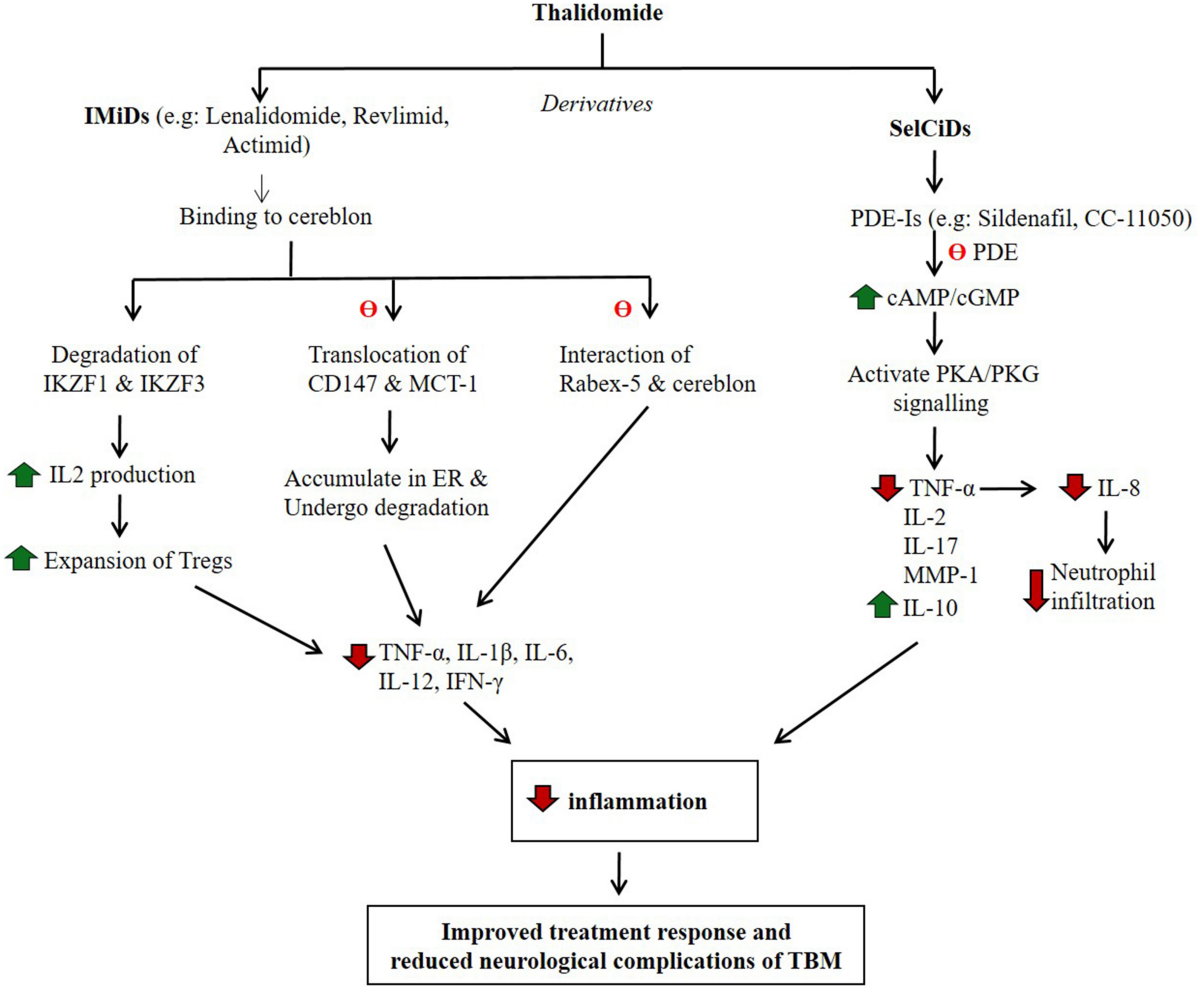
Potential mode of action of thalidomide and its derivatives in regulating host inflammatory response. IMiDs and SelCiDs are derivatives of thalidomide with anti-inflammatory properties. However, compounds of these two classes of anti-inflammatory agents function differentially as shown. Red arrow indicates down-regulation and green arrow indicate up-regulation. θ indicates inhibition of the pathway.

In contrast to IMiDs, SelCIDs selectively inhibit TNF-α production and do not stimulate lymphocyte proliferation; they have minimal effect on the production of other pro-inflammatory monocyte cytokines (Gordon and Goggin, 2003). The SelCIDs inhibit host phosphodiesterase-4 (PDE-4), an enzyme that hydrolyzes cyclic adenosine and guanosine monophosphates (cAMP and cGMP) to yield AMP and GMP, respectively (Figure 2). Both cAMP and cGMP transduce signals by interacting with protein kinase A (PKA) and PKG, respectively, and thereby modulate many physiological functions, including cell proliferation and differentiation, inflammation, and several metabolic pathways (Keravis and Lugnier, 2012). Inhibition of PDE4, the predominant PDE of monocytes and macrophages, results in dampening of the overly aggressive inflammatory responses in diverse diseases, including chronic obstructive pulmonary disease (COPD), asthma, pulmonary fibrosis, psoriatic arthritis, pulmonary TB, and others (Maurice et al., 2014). In both the mouse and rabbit models of pulmonary TB, administration of adjunctive PDE4 inhibitors (PDE4i), including CC-3052 and CC-11050, significantly reduced the bacillary load, macrophage activation, and the expression of pro-inflammatory molecules in the lungs (Koo et al., 2011; Subbian et al., 2011a,b, 2016a,b). Animals treated with PDE4i in combination with antibiotics showed accelerated clearance of Mtb infection, reduced fibrosis and reduction in lung granuloma size and numbers (Koo et al., 2011; Subbian et al., 2011a,b, 2016a,b). Similarly, another study reported that the PDE4i roflumilast and rolipram decreased the production of TNF-α, IL-6, CXCL1, and CXCL2/3, thus controlling neutrophil infiltration and subsequent lung inflammation in a rabbit model of pulmonary TB (Konrad et al., 2015). The efficacy of PDE4i, while evaluated extensively in preclinical models as well as in humans with newly diagnosed pulmonary TB and patients with ENL, has not been tested as adjunctive therapy for TBM.

## Conclusion

Since TBM continues to have an unacceptably high morbidity and mortality rate, particularly in young children, there is an urgent need for new, efficient adjunctive therapies for this devastating manifestation of TB. Results from the published literature suggest that the rabbit model of TBM can provide a useful tool for expanding our understanding of the pathologic process of CNS infection and disease. This preclinical model can facilitate the selection of optimal candidate adjunctive therapies to control the disease and improve the outcome of Mtb infection in the CNS of humans. The safety profile of PDE4i, described in this review, and their selective inhibition of monocyte-derived TNF-α, identify them as potantially efficacious adjunctive drugs to be used with antibiotics for the successful treatment of human TBM.

## Author Contributions

CV and SS conceived the concept. GK and SS designed the framework. RK, PS, AK, CV, GK, and SS wrote the manuscript. CV, GK, and SS edited the manuscript. All authors have read and agreed to publish the manuscript.

### Conflict of Interest

GK was a member of the board of directors of Celgene corporation from 1998-2018. The remaining authors declare that the research was conducted in the absence of any commercial or financial relationships that could be construed as a potential conflict of interest.
